# Enzymatic optimization for enhancing earthworm peptide yields and biological activities on antioxidant, cytotoxic against HT-29 and HepG2 cancer cells, and antimigration

**DOI:** 10.3389/fbioe.2026.1957959

**Published:** 2026-10-13

**Authors:** Pimphan Wasunan, Mongkol Thirabunyanon

**Affiliations:** Program in Biotechnology, Faculty of Science, Maejo University, Chiang Mai, Thailand

**Keywords:** antimigration, antioxidant, cytotoxicity, earthworm, HepG2, HT-29, peptides, anticancer

## Abstract

The purposes of this study were to investigate the antioxidant, cytotoxic against HT-29 and HepG2 cancer cells, and antimigratory activities of peptide fractions derived from *Amynthas arenulus* earthworm. Earthworm proteins were hydrolyzed with alcalase using response surface methodology. The optimal conditions resulted in a maximum degree of hydrolysis of 61.25% at an enzyme-to-substrate ratio of 1.5% (v/v) and a hydrolysis time of 2 h. Following simulated gastrointestinal digestion, gastric and intestinal recoveries of the peptides were 56.83% and 90.10%, respectively. The 10–30 kDa peptide fraction exhibited antioxidant activity, with estimated IC_50_ values of 3.18 and 0.79 mg/mL for DPPH and ABTS assays, respectively. FRAP activity was also exhibited. The 10–30 kDa peptide fraction showed antiproliferative activity against HT-29 and HepG2 cells, with IC_50_ values of 0.634 and 0.761 mg/mL, respectively, whereas it was not cytotoxic against non-cancerous cells of L929 fibroblast cells. This peptide fraction was trigger apoptosis-associated cellular changes observed using inverted light microscopy, AO/PI and DAPI staining, DNA fragmentation, and scanning electron microscopy. Relative cell migration values in HT-29 and HepG2 cells were 67.34% and 70.29%, respectively which compared to 100% of control. As resultants, this peptide fraction might be considered for applied in medical, nutraceutical food, and bioprocessing industries.

## 1 Introduction

Human colorectal cancer is the third most common cancer in terms of incidence and the second leading cause of cancer-related mortality worldwide. According to GLOBOCAN 2024, approximately 2.04 million new colorectal cancer cases and 917,895 deaths were estimated globally in 2024 (Sung et al., 2026). Likewise, hepatocellular carcinoma is the predominant form of primary liver cancer. Liver cancer ranked seventh in incidence and third in cancer-related mortality globally in 2024, with approximately 843,045 new cases and 732,489 deaths (Sung et al., 2026).

Terrestrial earthworms have gained popularity as important nutritional and medicinal agents. Natural bioactive compounds obtained from terrestrial earthworms are of particular interest to researchers. Earthworms have been the focus of continuous biomedical research. In particular, they have shown potential as anti-inflammatory (Huang et al., 2016; Afshar et al., 2022), antimicrobial (Mustafa et al., 2022), antioxidant (Wasunan et al., 2022), and anticancer (Shafi and Faleh, 2019) agents. *Amynthas* sp. earthworms have increasingly attracted attention because of their high peptide content, obtained mainly from earthworm paste (Chen et al., 2010), earthworm powder (Chang et al., 2011; Wasunan et al., 2022), and coelomic fluid (Mustafa et al., 2022). Proteins are polypeptides composed of amino acids connected by peptide bonds.

Animal proteins have different properties depending on the animal species. When proteins are hydrolyzed with enzymes, they produce different peptide profiles depending on the enzymatic hydrolysis conditions, including the enzyme-to-substrate ratio, pH, temperature, and hydrolysis time (Jamil et al., 2016). In studies of protein hydrolysates, researchers commonly use response surface methodology (RSM) to determine optimal hydrolysis conditions (Wasswa et al., 2008; Jamil et al., 2016; Rodrigues et al., 2017; Hunsakul et al., 2022). RSM is a statistical and mathematical approach that can be used to optimize protein hydrolysis conditions (Hunsakul et al., 2022). Following hydrolysis, the yield and degree of hydrolysis are commonly evaluated (Jamil et al., 2016).

Alcalase®, derived from *Bacillus licheniformis*, is a serine endopeptidase. Alcalase can hydrolyze proteins with broad specificity, particularly peptide bonds involving large uncharged residues (Setthaya et al., 2021). It has been shown to cleave peptide bonds on the carboxyl side of Glu, Met, Leu, Tyr, Lys, and Gln residues (Adamson and Reynolds, 1996). Alcalase exhibits broad cleavage specificity and high hydrolytic activity. Consequently, Alcalase-catalyzed protein hydrolysis has a high propensity to produce hydrolysates rich in small peptides (Tacias-Pascacio et al., 2020).

Alcalase-pretreated bioactive peptides can be derived from several dietary protein sources. Bioactive peptides have been incorporated into nutraceuticals (Moldes et al., 2017) and functional foods (Haque et al., 2008) because of their various biological activities, including antioxidant activity from spotted babylon snail (Petsantad et al., 2020), anti-inflammatory activity from mealworm larvae, adult crickets, and silkworm pupae (Yoon et al., 2019), antidiabetic activity from crickets (*Gryllodes sigillatus*) (Hall et al., 2018), angiotensin-converting enzyme inhibitory activity from *Stichopus horrens* (Forghani et al., 2012), antihypertensive activity from olive flounder *(Paralichthys olivaceus*) (Oh et al., 2020), antimicrobial activity from *Pheretima posthuman* earthworm (Hussain et al., 2021), and anticancer activity from silkworm pupae (Cermeño et al., 2022).

Furthermore, *Amynthas corticis* earthworm has been reported to exhibit anticoagulant, antibacterial, and antioxidant activities (Mustafa et al., 2022). *Pheretima aspergillum* (homotypic synonym; *Amynthas aspergillus*) earthworm has also been reported to enhance PC-12 cell proliferation (Chen et al., 2010). However, studies on Alcalase-pretreated earthworm proteins, particularly those derived from *Amynthas arenulus*, remain limited. Therefore, this study focused on the biological activities of peptide fractions derived from *A. arenulus* earthworm proteins. This species was identified in northeastern Thailand (Wasunan et al., 2024). RSM was used to optimize the protein hydrolysis conditions, and the resulting peptide material was evaluated following simulated gastrointestinal digestion. The peptide fractions were subsequently evaluated for antioxidant, *in vitro* cytotoxic against human colorectal adenocarcinoma (HT-29) and hepatocellular carcinoma (HepG2) cells, and antimigratory activities.

## 2 Materials and methods

### 2.1 Protein extraction of *A. arenulus* earthworm

Isoelectric point precipitation was used to separate proteins from *A. arenulus* earthworm according to Lee et al. (2016). In brief, defatted earthworm powder was solubilized in an alkaline solution (pH 10.0) at a ratio of 1:10 (w/v) at 50 °C for 1 h and then centrifuged at 2,701 × g to collect the supernatant. The proteins were precipitated by adjusting the pH to 4.0, followed by centrifugation and lyophilization.

### 2.2 Optimal hydrolysis of *A. arenulus* earthworm protein

Protein isolates were hydrolyzed with Alcalase® derived from *B. licheniformis* (2.4 U/g; Sigma-Aldrich, St. Louis, MO, United States) using a central composite design (CCD) of response surface methodology (RSM) (Rodrigues et al., 2017). Isolated proteins were prepared in phosphate buffer (1% (w/v), pH 8.0). Two parameters were evaluated: the enzyme-to-substrate (E/S) ratio (1%–2% (v/v)) and hydrolysis time (1–3 h). The experiments were carried out at 60 °C with a shaking speed of 200 rpm. The experimental design, codes, units, and factor levels used for optimization are shown in Table 1. Table 2 presents the 13 experimental treatments generated by the CCD. The resulting protein hydrolysates were evaluated for yield and the trichloroacetic acid (TCA)-soluble peptide index (DH–TCA).

**TABLE 1:** The experimental parameters, code, unit, and level factors for optimization.

Parameter	Coding	Unit	Levels		
			−1	0	+1
E/S ratio	A	% v/v	1	1.5	2
Time	B	hour	1	2	3

**TABLE 2:** Treatments created using the central composite design for optimum hydrolysis.

​	Parameters		Protein hydrolysate (g/g protein)		Degree of hydrolysis (%)	
Treatments	A	B	Actual value	Predicted value	Actual value	Predicted value
1	0	0	12.41	13.04	52.41	57.63
2	−1	−1	15.25	15.52	50.21	51.48
3	1	−1	14.29	15.27	44.64	43.24
4	2.21	0	16.57	16.02	39.54	42.12
5	0	0.59	15.57	14.70	50.34	50.15
6	0	0	11.20	13.04	53.43	57.63
7	1	1	14.03	13.86	48.70	46.04
8	0	0	13.45	13.04	60.70	57.63
9	0	3.41	10.19	10.95	43.79	45.37
10	0	0	13.72	13.04	61.60	57.63
11	−1	1	12.50	11.62	41.92	41.93
12	0.79	0	14.16	14.61	46.24	45.05
13	0	0	14.41	13.04	59.99	57.63

### 2.3 Determination of the TCA-soluble peptide index

The extent of enzymatic hydrolysis was estimated using the 10% trichloroacetic acid, TCA-soluble peptide method, with modifications based on Hoyle and Merritt (1994). This method provides an operational estimate of proteolysis based on the proportion of relatively small peptide products that remain soluble after precipitation of larger proteins with TCA. An aliquot of the protein hydrolysate was mixed at a 1:1 (v/v) ratio with 20% (w/v) TCA to obtain a final TCA concentration of 10% (w/v). The mixture was incubated at room temperature for 30 min and subsequently centrifuged at 2,701 × g for 15 min. The peptide content of the TCA-soluble supernatant was determined using the Lowry method (Lowry et al., 1951). Total protein content of the hydrolysate was determined using the same method. The TCA-soluble peptide index was calculated as follows: DH–TCA (%) = [TCA-soluble peptide content/total protein content] × 100 (Silvestre et al., 2013). The resulting DH–TCA value represents the proportion of TCA-soluble peptide material and provides an indirect operational index of proteolysis rather than a direct measurement of peptide-bond cleavage.

### 2.4 *In vitro* gastrointestinal digestion and recovery of peptide material

Protein hydrolysates from *A. arenulus* earthworms were subjected to simulated gastrointestinal digestion according to (Kannan et al., 2008), with modifications. For the simulated gastric phase, the protein hydrolysates were incubated in a gastric solution containing pepsin (≥250 units/mg solid; CAS No. 9001-75-6; Sigma-Aldrich, St. Louis, MO, United States) and sodium chloride at concentrations of 0.32% and 0.20% (w/v), respectively. The pH was adjusted to 2.0, and the mixture was incubated at 37 °C for 2 h with continuous shaking at 120 rpm. After gastric digestion, pepsin activity was terminated by adjusting the pH to 7.2. The mixture was then centrifuged at 2,701 × *g* for 30 min at 4 °C. The soluble fraction was collected, lyophilized, and weighed. Gastric recovery was calculated as follows:

$$\text{Gastric recovery }\left(\%\right)=\left(\text{Wg }/\;W0\right)\times 100$$

where *Wg* represents the dry weight of the lyophilized soluble peptide material recovered after simulated gastric digestion, and *W0* represents the initial dry weight of the protein hydrolysate subjected to gastric digestion.

For the simulated intestinal phase, a defined amount of the lyophilized peptide material recovered after gastric digestion was subjected to simulated intestinal digestion. The intestinal solution contained 0.1% (w/v) pancreatin (CAS No. 8049-47-6; Sigma-Aldrich, St. Louis, MO, United States), 0.68% (w/v) potassium phosphate monobasic, and 7.7% (v/v) of 0.2 N sodium hydroxide. The pH was adjusted to 8.0, and the mixture was incubated at 37 °C for 2 h with continuous shaking at 120 rpm. Pancreatin activity was terminated by heating the mixture at 85 °C for 10 min. The mixture was subsequently centrifuged at 2,701 × g for 30 min at 4 °C. The soluble fraction was collected, lyophilized, and weighed. Intestinal recovery was calculated as follows:

$$\text{Intestinal recovery }\left(\%\right)=\left(\text{Wi }/\text{ Win}\right)\times 100$$

where *Wi* represents the dry weight of the lyophilized soluble peptide material recovered after simulated intestinal digestion, and *Win* represents the dry weight of the gastric-digested peptide material initially subjected to intestinal digestion.

The gastric and intestinal recovery values represent the gravimetric recovery of lyophilized soluble peptide material after each simulated digestion phase, rather than the proportion of individual intact peptides resistant to gastrointestinal proteolysis. The peptide material recovered after simulated gastrointestinal digestion was lyophilized and stored at −20 °C until further fractionation and biological activity analyses.

### 2.5 Fractionation of the peptide fraction recovered after simulated gastrointestinal digestion by ultrafiltration

The peptide material recovered after simulated gastrointestinal digestion was fractionated by ultrafiltration according to apparent molecular-weight ranges. Ultrafiltration was performed using membranes with molecular-weight cutoffs (MWCOs) of 50, 30, and 10 kDa (GE Healthcare Bio-Sciences AB, Sweden). The recovered peptide material was first subjected to ultrafiltration using a 50-kDa MWCO membrane. The fraction passing through the 50-kDa membrane was subsequently fractionated using a 30-kDa MWCO membrane, followed by a 10-kDa MWCO membrane. Based on the sequential ultrafiltration procedure, three peptide fractions with apparent molecular-weight ranges of 10–30 kDa, 30–50 kDa, and >50 kDa were obtained. The resulting peptide fractions were lyophilized and stored at −20 °C until further biological activity analyses. Molecular-weight cutoff-based ultrafiltration provides an operational separation according to apparent molecular size and does not identify the individual peptide sequences or molecular species present in each fraction.

### 2.6 Antioxidant activity

#### 2.6.1 DPPH radical-scavenging activity assay

The DPPH radical-scavenging activity of the peptide fractions was determined according to (Mungmuang et al., 2025; Senphan et al., 2026), with modifications. The peptide fractions were mixed with 0.1 mM DPPH radical solution prepared in methanol (Sigma-Aldrich, United States) at a ratio of 1:4 (v/v). The reaction mixtures were incubated at room temperature for 30 min, and the absorbance was measured at 517 nm using a microplate reader (SPECTROstar Nano, BMG LABTECH, Germany). α-Tocopherol was experimentally tested as a positive control under the same assay conditions. The DPPH radical-scavenging activities were calculated using the following Equation 1.

#### 2.6.2 ABTS radical-scavenging activity assay

The ABTS radical-scavenging activity of the peptide fractions was determined according to (Petsantad et al., 2020), with modifications. The ABTS radical cation (ABTS^•+^) solution was generated by mixing 7 mM ABTS (Sigma-Aldrich, Germany) with 2.45 mM potassium persulfate (Ajax Finechem, Australia) and incubating the mixture at room temperature in the dark for 16 h. Prior to use, the ABTS^•+^ solution was diluted with methanol to obtain an absorbance of 0.700 ± 0.020 at 734 nm. The peptide fractions were then mixed with the diluted ABTS^•+^ solution at a ratio of 1:30 (v/v) and incubated at room temperature for 10 min. The absorbance was measured at 734 nm using a microplate reader. α-Tocopherol was experimentally tested as a positive control under the same assay conditions. The ABTS radical-scavenging activities were calculated using the following Equation 1.

#### 2.6.3 FRAP assay

The ferric-reducing antioxidant power (FRAP) of the peptide fractions was determined according to (Farsani et al., 2021), with modifications. The FRAP reagent was freshly prepared by mixing 300 mM acetate buffer (pH 3.6), 10 mM TPTZ in 40 mM HCl, and 20 mM ferric chloride at a ratio of 10:1:1 (v/v/v). The peptide fractions were mixed with the FRAP reagent at a ratio of 1:30 (v/v) and incubated at room temperature for 10 min. The absorbance was measured at 593 nm using a microplate reader. A corresponding sample blank was measured for each sample, and the blank-corrected FRAP response was calculated as: ΔA593 = A593 (sample reaction) − A593 (sample blank). The results were expressed as blank-corrected absorbance at 593 nm (ΔA593, AU), with higher ΔA593 values indicating greater ferric-reducing activity. α-Tocopherol was experimentally tested as a positive control under the same assay conditions and analyzed using the same blank-correction procedure.

#### 2.6.4 Calculation of antioxidant activity

The DPPH and ABTS radical-scavenging activities were calculated using the following equation:

$$\text{Radical}‐\text{scavenging activity }\left(\%\right)=\left[\left(A_{\text{control}}\;‐\;A_{\text{sample}}\right)\;/\;A_{\text{control}}\right]\times 100$$ (1)

where *A*_control_ is the absorbance of the control reaction and *A*_sample_ is the absorbance of the sample reaction. The IC_50_ value was defined as the concentration required to scavenge 50% of DPPH or ABTS radicals and was estimated from the corresponding concentration-response curves.

### 2.7 Cell culture conditions

The human colorectal adenocarcinoma (HT-29) and human hepatocellular carcinoma (HepG2) cell lines were purchased from the American Type Culture Collection (ATCC). The cells were cultured in Dulbecco’s Modified Eagle Medium (DMEM) supplemented with 10% fetal bovine serum (FBS), 1% 100× MEM non-essential amino acids (NEAA), and 1% penicillin–streptomycin (10,000 U/mL penicillin and 10,000 μg/mL streptomycin); all reagents were obtained from Gibco, United States. The cells were maintained at 37 °C in a humidified incubator containing 5% CO_2_ (Forma Scientific, Model 3111, United States).

### 2.8 MTT assay

The colorimetric MTT assay [3-(4,5-dimethylthiazol-2-yl)-2,5-diphenyltetrazolium bromide; Sigma-Aldrich, United States] was used to evaluate the antiproliferative activity of the peptide fractions against HT-29 and HepG2 cells (Chatnarin and Thirabunyanon, 2024). HT-29 or HepG2 cells were detached using 0.5% trypsin-EDTA (Gibco, United States). One hundred microliters of the cell suspension (5 × 10^4^ cells/mL) were seeded into 96-well plates (Nunc, China) and incubated for 24 h at 37 °C in a humidified atmosphere containing 5% CO_2_. Next, 100 µL of the peptide fractions (final concentrations of 0.1–0.8 mg/mL) was added and incubated for an additional 24 h under the same conditions. MTT solution (10 μL, 0.5 mg/mL) was then added, followed by incubation for 4 h at 37 °C in 5% CO_2_. The resulting formazan crystals were solubilized with 100 µL of DMSO (Sigma-Aldrich, United States) and incubated for 10 min. Absorbance was measured at 595 nm using a microplate reader. The experiments were carried out independently three times, with each experiment conducted in triplicate. Untreated cells served as the control. The percentage of cell death, estimated from the MTT assay, was calculated using the following equation:

$$\text{Cell death }\left(\%\right)=\left[1\;‐\;\left(A_{\text{sample}}\;/\;A_{\text{control}}\right)\right]\times 100$$

where *A*_sample_ and *A*_control_ represent the absorbance values of the treated and untreated control cells, respectively.

### 2.9 Determination of morphological features under an inverted light microscope

HT-29 and HepG2 cells were treated with 500 µL of the 10–30 kDa peptide fraction at concentrations corresponding to their respective IC_50_ values (0.634 mg/mL for HT-29 cells and 0.761 mg/mL for HepG2 cells). After treatment for 24 h, the cells were carefully washed twice with PBS. Untreated cells and cells treated with 1 nM paclitaxel served as the control and positive control, respectively. Cellular morphological features were examined under an inverted light microscope (Olympus, Japan) according to Thirabunyanon et al. (2024), with modifications.

### 2.10 Cytotoxicity in non-cancerous cells

L929 mouse fibroblast cells were used as a non-cancerous cell model to examine the cytotoxicity of the 10–30 kDa peptide fraction. The cells were exposed to the fraction at concentrations of 0.634 and 0.761 mg/mL, corresponding to the IC_50_ values previously determined for HT-29 and HepG2 cells, respectively. Untreated cells maintained in DMEM served as the negative control, while ascorbic acid was included as a reference treatment. After incubation, cell viability was assessed using the MTT assay (Huang et al., 2020). The viability of the treated cells was expressed relative to that of the untreated control using the following equation: The viability of treated cells was expressed relative to that of the untreated control using the following equation:

$$\text{Cell viability }\left(\%\right)=\left(A_{\text{sample}}\;/\;A_{\text{control}}\right)\times 100.$$

where *A*_sample_ and ***A***_control_ represent the absorbance values of treated and untreated cells, respectively.

### 2.11 Assessment of apoptosis-associated morphological features using acridine orange/propidium iodide double staining

AO/PI double staining was used to evaluate apoptosis-associated morphological changes in HT-29 and HepG2 cells following treatment with the 10–30 kDa peptide fraction. Acridine orange (AO) and propidium iodide (PI) (Sigma-Aldrich, United States) were used according to (Srikham et al., 2021), with modifications. Briefly, cells were seeded at 5 × 10^4^ cells/mL (500 µL) in an 8-well chamber slide (SPL, Korea). HT-29 and HepG2 cells were treated with 500 µL of the 10–30 kDa peptide fraction at concentrations of 0.634 and 0.761 mg/mL, respectively, corresponding to their previously determined IC_50_ values. The cells were incubated at 37 °C in a humidified atmosphere containing 5% CO_2_ for 24 h. After treatment, the cells were carefully rinsed with PBS and stained for 10 min with 500 µL of an AO/PI solution prepared by mixing AO and PI solutions (each 10 μg/mL in PBS) at a ratio of 1:1 (v/v). The cells were then washed with PBS. Apoptosis-associated morphological features were examined under an inverted fluorescence microscope (Nikon Eclipse Ti-U, Japan). Untreated cells served as the control, whereas cells treated with 1 nM paclitaxel served as the positive control.

### 2.12 Assessment of nuclear chromatin condensation using DAPI staining

DAPI (4′,6-diamidino-2-phenylindole; Sigma, United States) staining was used to examine nuclear morphological changes, including chromatin condensation and nuclear fragmentation (Salmanzadeh et al., 2018). Briefly, HT-29 and HepG2 cells were seeded at 5 × 10^4^ cells/mL (500 µL) in an 8-well chamber slide and incubated for 24 h at 37 °C in a humidified atmosphere containing 5% CO_2_. HT-29 cells were then treated with 500 µL of the 10–30 kDa peptide fraction at 0.634 mg/mL, whereas HepG2 cells were treated with 500 µL of the same fraction at 0.761 mg/mL, corresponding to their respective IC_50_ values. The cells were further incubated for 24 h under the same conditions. After treatment, the cells were gently rinsed twice with PBS and stained with freshly prepared DAPI solution (10 μg/mL) for 10 min in the dark. The cells were then gently rinsed twice with PBS. Nuclear morphological changes, including chromatin condensation and nuclear fragmentation, were examined using an inverted fluorescence microscope. Untreated cells served as the control, whereas cells treated with 1 nM paclitaxel served as the positive control.

### 2.13 Assessment of DNA fragmentation by agarose gel electrophoresis

DNA fragmentation was examined as an additional indicator of apoptosis-associated cellular changes (Srikham and Thirabunyanon, 2022). Briefly, 1 mL of HT-29 or HepG2 cell suspension (5 × 10^4^ cells/mL) was seeded into 6-well plates (SPL, Korea) and incubated for 24 h at 37 °C in a humidified atmosphere containing 5% CO_2_. HT-29 cells were then treated with 500 µL of the 10–30 kDa peptide fraction at 0.634 mg/mL, whereas HepG2 cells were treated with 500 µL of the same fraction at 0.761 mg/mL, corresponding to their respective IC_50_ values. The cells were further incubated for 24 h under the same conditions. Untreated cells served as the control, whereas cells treated with 1 nM paclitaxel served as the positive control. The cells were collected and centrifuged at 13,709 × g for 5 min. DNA was extracted from the resulting cell pellets according to the manufacturer’s protocol using a DNA extraction kit (Tiangen, Taiwan). The extracted DNA was electrophoresed on a 1.5% agarose gel (Vivantis, United States) in 0.5× Tris-borate-EDTA (TBE) buffer (Bio Basic, Canada) at 80 V for 85 min. GeneRuler 100 bp Plus DNA Ladder (Thermo Fisher Scientific, Lithuania) was used as the molecular-size marker. The DNA banding patterns were subsequently visualized using an UltraSlim LED illuminator (California, United States).

### 2.14 Scanning electron microscope

Scanning electron microscopy (SEM) was performed to examine cell-surface morphological features (Chatnarin and Thirabunyanon, 2023 **)**. Briefly, 1 mL of HT-29 or HepG2 cell suspension (5 × 10^4^ cells/mL) was seeded into 6-well plates and incubated for 24 h at 37 °C in a humidified atmosphere containing 5% CO_2_. HT-29 and HepG2 cells were then treated with 1 mL of the 10–30 kDa peptide fraction at concentrations of 0.634 and 0.761 mg/mL, respectively, corresponding to their previously determined IC_50_ values. The cells were further incubated for 24 h under the same conditions. Untreated cells and cells treated with 1 nM paclitaxel served as the control and positive control, respectively. The cells were collected by centrifugation at 4,319 × g for 1 min, and the resulting cell pellets were fixed with 3% glutaraldehyde for 24 h at 4 °C. The cells were dehydrated sequentially in increasing concentrations of ethanol (30%, 50%, 70%, 80%, 90%, 95%, and 100%), with each dehydration step performed for 10 min (Ji et al., 2019). Cells immersed in absolute ethanol were subsequently dried using critical-point drying (CPD). The dried cells were mounted on stubs using double-sided carbon adhesive film and coated with an 8-nm-thick layer of gold at 30 mA using a high-vacuum coater. Cell-surface morphology was examined using a high-vacuum scanning electron microscope (HV-SEM; TESCAN Vega III, Czech Republic) at an accelerating voltage of 7 kV. Images were acquired using secondary electron (SE) detection.

### 2.15 Scratch assay for cell migration

The scratch assay was performed as previously described by Liang et al. (2007). HT-29 and HepG2 cells (5 × 10^4^ cells/mL) were seeded in 24-well plates (SPL, Korea) and cultured until confluent monolayers were obtained. A straight scratch was created using a sterile 0.2 mL pipette tip, and the cells were gently washed with PBS to remove detached cells and debris. HT-29 and HepG2 cells were treated with the 10–30 kDa peptide fraction at 0.634 and 0.761 mg/mL, respectively, corresponding to the IC_50_ values determined by the MTT assay. Untreated cells served as the control, whereas cells treated with 1 nM paclitaxel served as the positive control. Images of the scratched area were acquired at 0 and 24 h using an inverted light microscope at ×200 magnification. Three independent experiments were performed, each in triplicate. The scratched areas at 0 h (A_0_) and 24 h (A_24_) were quantified using ImageJ software (version 1.53k; National Institutes of Health, Bethesda, MD, United States). Cell migration was calculated as follows:

$$\text{Cell migration }\left(\%\right)=\left[\left(A_{0}\;‐\;A_{24}\right)\;/\;A_{0}\right]\times 100$$

where A_0_ and A_24_ represent the scratched areas measured at 0 and 24 h, respectively. Because the peptide fraction was tested at its IC_50_ concentration, the observed migration values may reflect combined effects on cell proliferation and cell movement. Therefore, the results were interpreted as apparent cell migration under the tested conditions rather than as evidence of a specific antimigratory effect.

### 2.16 Statistical evaluation

Results were expressed as mean ± standard deviation (SD). Statistical analyses were performed using IBM SPSS Statistics software (version 28.0). One-way or two-way analysis of variance (ANOVA) was used as appropriate. Duncan’s multiple range test was used for *post hoc* comparisons following one-way ANOVA. Differences were considered statistically significant at *p* < 0.05.

## 3 Results

### 3.1 Effect of hydrolysis conditions on protein hydrolysate yield

Protein hydrolysate yield was evaluated to determine the effects of the enzyme-to-substrate (E/S) ratio and hydrolysis time. The overall model was not statistically significant (*p* > 0.05), with a coefficient of determination (R^2^) of 0.7310 (Table 3). The fitted relationship between the E/S ratio and hydrolysis time is described by the coded equation shown in Equation 2.

**TABLE 3:** ANOVA model for yield productive optimization as influenced by alcalase

Source of variance	Sum of squares	Degrees of freedom	Mean square	*F* value	*P* value
Model	27.07	5	5.41	3.80	0.0552
A-E/S	1.98	1	1.98	1.39	0.2769
B-time	14.09	1	14.09	9.90	0.0162
AB	1.55	1	1.55	1.09	0.3314
A^2^	9.00	1	9.00	6.32	0.0401
B^2^	0.0771	1	0.0771	0.0541	0.8227
Residual	9.96	7	1.42	​	​
Lack of fit	3.67	3	1.22	0.7784	0.5642
Pure error	6.29	4	1.57	​	​
Cor total	37.04	12	​	​	​
Std. Dev	1.19	​	*R* ^2^	0.7310	​
Mean	13.67	​	Adjusted *R* ^2^	0.5389	​
C.V. %	8.72	​	Predicted *R* ^2^	0.0296	​
​	​	​	Adeq precision	6.2500	​

The contour (Figure 1A) and response surface plots (Figure 1B) indicated that the protein hydrolysate yield ranged from 10.19 g/g protein to 16.57 g/g protein (Table 2). The highest protein hydrolysate yield was observed in treatment 4, with an E/S ratio of 2.21% (v/v) and a hydrolysis time of 2 h, resulting in a yield of 16.57 g/g protein. In contrast, treatment 9, performed at an E/S ratio of 1.5% (v/v) for 3.41 h, produced the lowest protein hydrolysate yield of 10.19 g/g protein.

$$\text{Protein hydrolysate yield }\left(\%\right)=13.04+0.49A\;‐\;1.33B+0.62\text{AB}+1.14A^{2}\;‐\;0.11B^{2}$$ (2)

**FIGURE 1:**
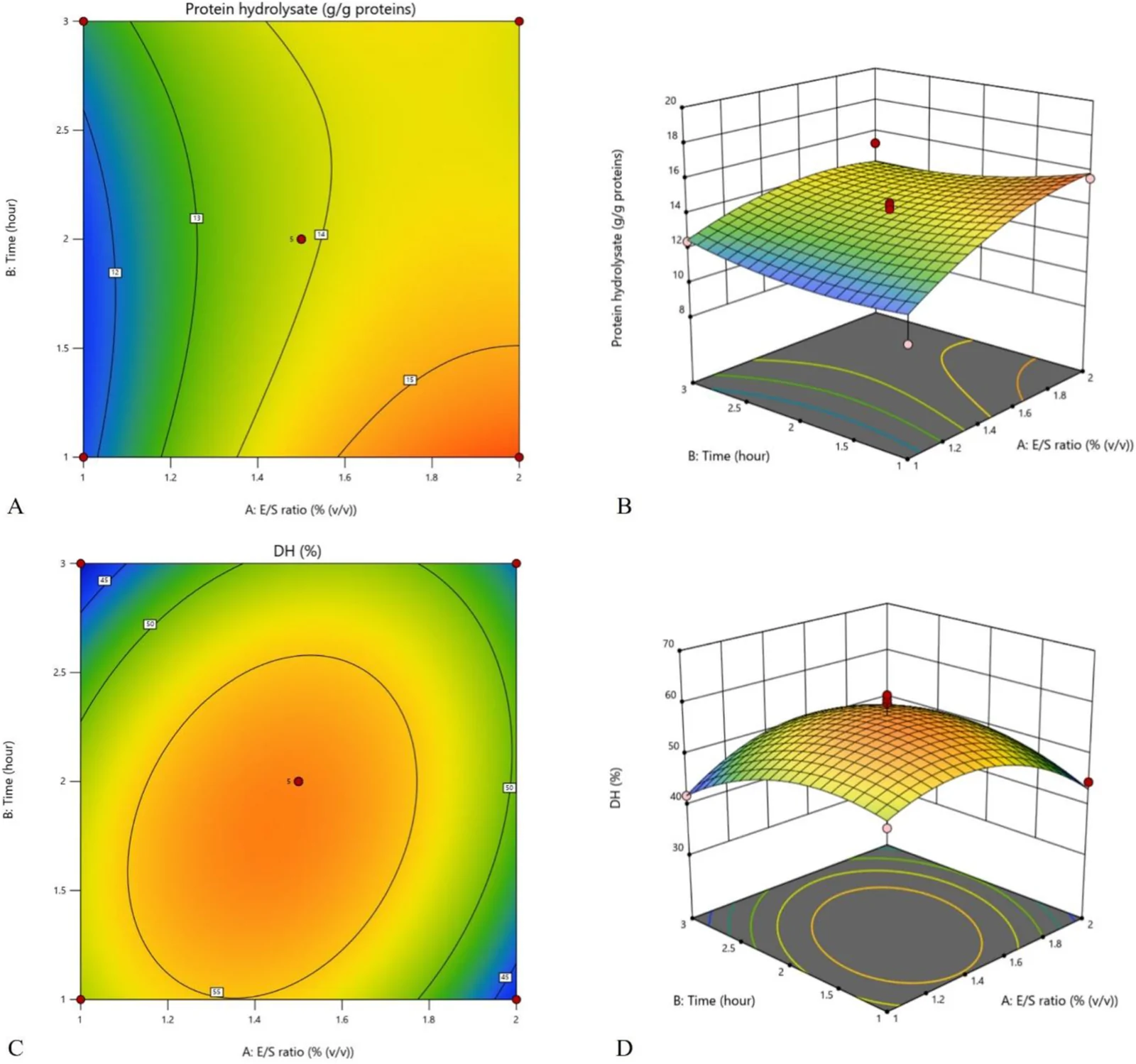
Protein hydrolysate yield as a function of the enzyme-to-substrate (E/S) ratio and hydrolysis time, presented as a contour plot **(A)** and a response surface plot **(B)**. TCA-soluble peptide index (DH-TCA) as a function of the enzyme-to-substrate (E/S) ratio and hydrolysis time, presented as a contour plot **(C)** and a response surface plot **(D)**.

### 3.2 Effect of optimal hydrolysis on the degree of hydrolysis

The TCA-soluble peptide index (DH−TCA) was used as an indirect indicator of the extent of enzymatic protein hydrolysis. Higher DH−TCA values indicate a greater proportion of relatively small peptide products remaining soluble in 10% TCA. The DH−TCA was analyzed using ANOVA to evaluate the effects of the enzyme-to-substrate (E/S) ratio and hydrolysis time. Model terms with p-values <0.05 were considered statistically significant. The model showed an R^2^ value of 0.8445 (Table 4). The fitted relationship between the E/S ratio and hydrolysis time is described by the coded equation presented in Equation 3.

**TABLE 4:** ANOVA model for degree of hydrolysis (DH) optimization as influenced by alcalase

Source of variance	Sum of squares	Degrees of freedom	Mean square	*F* value	*P* value
Model	526.51	5	105.30	7.60	0.0095
A-E/S	8.54	1	8.54	0.6166	0.4581
B-time	22.76	1	22.76	1.64	0.2407
AB	38.13	1	38.13	2.75	0.1410
A^2^	342.87	1	342.87	24.76	0.0016
B^2^	169.28	1	169.28	12.22	0.0100
Residual	96.95	7	13.85	​	​
Lack of fit	21.30	3	7.10	0.3755	0.7764
Pure error	75.64	4	18.91	​	​
Cor total	623.45	12	​	​	​
Std. Dev.	3.72	​	R^2^	0.8445	​
Mean	50.27	​	Adjusted R^2^	0.7334	​
C.V. %	7.40	​	Predicted R^2^	0.5674	​
​	​	​	Adeq precision	6.2076	​

The contour (Figure 1C) and response surface plots (Figure 1D) indicated model-predicted DH−TCA values ranging from 39.54% to 61.60% (Table 2). Experimentally, treatment 10 showed the highest DH−TCA value of 61.60% at an E/S ratio of 1.5% (v/v) and a hydrolysis time of 2 h, whereas treatment 4 showed the lowest DH−TCA value of 39.54% at an E/S ratio of 2.21% (v/v) and a hydrolysis time of 2 h.

$$\text{DH}‐\text{TCA }\left(\%\right)=57.63\;‐\;1.03A\;‐\;1.69B+3.09\text{AB }‐\;7.02A^{2}\;‐\;4.93B^{2}$$ (3)

### 3.3 Simulated gastrointestinal digestion, recovery of peptide material, and peptide fractionation

The peptide material showed a gastric recovery of 56.83% following simulated gastric digestion and an intestinal recovery of 90.10% following simulated intestinal digestion (Table 5). These values represent the gravimetric recovery of lyophilized soluble peptide material after each digestion phase rather than the proportion of intact peptides resistant to gastrointestinal proteolysis. The peptide material recovered after simulated gastrointestinal digestion was subsequently fractionated by ultrafiltration using membranes with molecular-weight cutoffs (MWCOs) of 50, 30, and 10 kDa. Three peptide fractions with apparent molecular-weight ranges of 10–30, 30–50, and >50 kDa were obtained.

**TABLE 5:** The percentage of protein hydrolysates from *A. arenulus* earthworms tolerating the mimic of gastrointestinal tract. The experiments were carried out in triplicate and independently (nd: no determined).

Initial protein hydrolysates (g)	Stomach tract (g)	Intestinal tract (g)	Peptides (%)
5	2.84 ± 0.21	nd	56.83
2.5	nd	2.25 ± 0.28	90.10

### 3.4 Antioxidant activity

The antioxidant activities of the peptide fractions were evaluated using DPPH, ABTS, and FRAP assays. The DPPH and ABTS assays were used to assess free radical-scavenging activity, whereas the FRAP assay was used to evaluate ferric-reducing activity. In the DPPH assay, the 10–30, 30–50, and >50 kDa peptide fractions exhibited radical-scavenging activity, with estimated IC_50_ values of 3.18, 4.08, and 2.49 mg/mL, respectively (Figure 2A). Among the three fractions, the >50 kDa fraction showed the lowest estimated IC_50_ value. α-Tocopherol, used as a positive control, exhibited an estimated IC_50_ value of 0.25 mg/mL under the same assay conditions. Thus, α-tocopherol showed a lower estimated IC_50_ value than the peptide fractions, suggesting greater DPPH radical-scavenging activity under the tested conditions.

**FIGURE 2:**
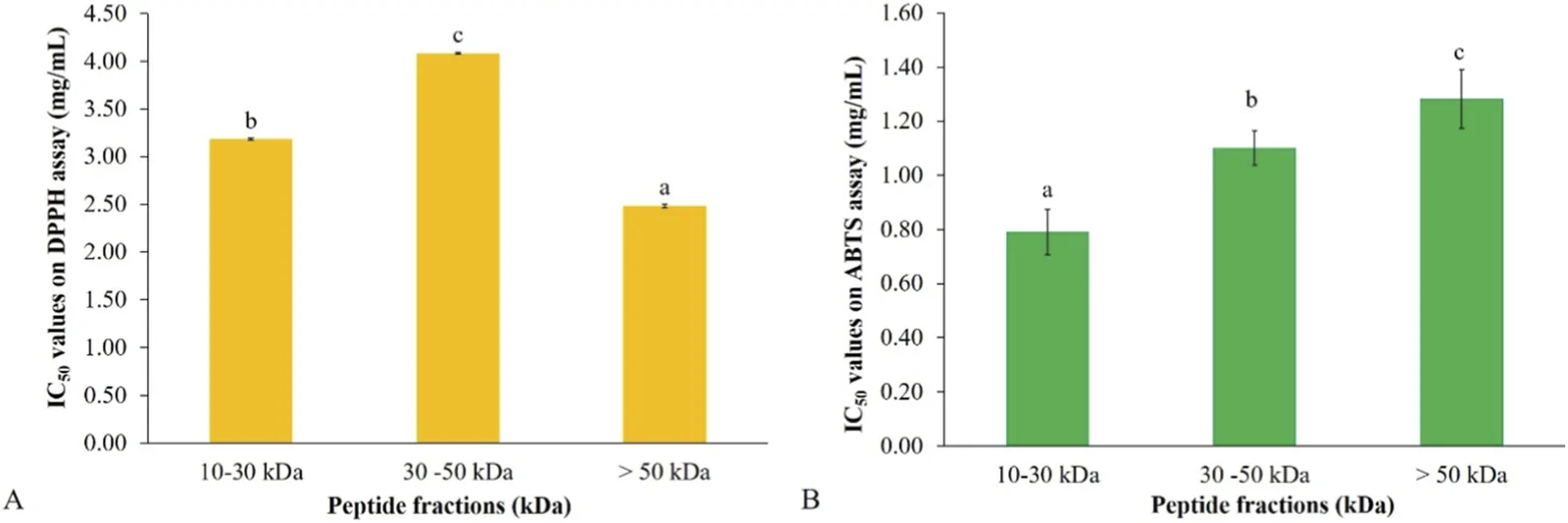
Antioxidant activities of the peptide fractions. DPPH radical-scavenging activity **(A)** and ABTS radical-scavenging activity **(B)**. Different lowercase letters (a–c) indicate significant differences among groups (*p* < 0.05).

For the ABTS assay, the estimated IC_50_ values of the 10–30, 30–50, and >50 kDa peptide fractions were 0.79, 1.10, and 1.28 mg/mL, respectively (Figure 2B). The 10–30 kDa fraction showed the lowest estimated IC_50_ value among the three fractions. α-Tocopherol, used as a positive control, showed an estimated IC_50_ value of 1.47 mg/mL under the same assay conditions. Thus, the three peptide fractions showed numerically lower estimated IC_50_ values than α-tocopherol, with the 10–30 kDa fraction showing the lowest value.

The FRAP assay was used to evaluate the ferric-reducing activity of the peptide fractions. The results were expressed as blank-corrected absorbance at 593 nm (ΔA593, AU), as shown in Figure 3. Across the tested concentrations (0.02–0.5 mg/mL), the 10–30, 30–50, and >50 kDa fractions exhibited ΔA593 values ranging from 1.020 to 1.046, 1.025 to 1.035, and 1.017 to 1.025 AU, respectively. Two-way ANOVA revealed no significant effects of molecular-weight fraction (p = 0.261), concentration (p = 0.539), or their interaction (p = 0.326). These results indicate that the three peptide fractions exhibited comparable ferric-reducing responses across the tested concentration range. α-Tocopherol, used as a positive control, exhibited ΔA593 values ranging from 0.680 to 1.092 AU across the tested concentrations. In separate comparisons with α-tocopherol, all three peptide fractions exhibited significantly higher ΔA593 values at 0.05 and 0.10 mg/mL (*p* < 0.05), whereas no significant differences were observed at 0.02, 0.20, or 0.50 mg/mL (*p* > 0.05). These findings indicate that the relative ferric-reducing responses of the peptide fractions and α-tocopherol depended on the tested concentration.

**FIGURE 3:**
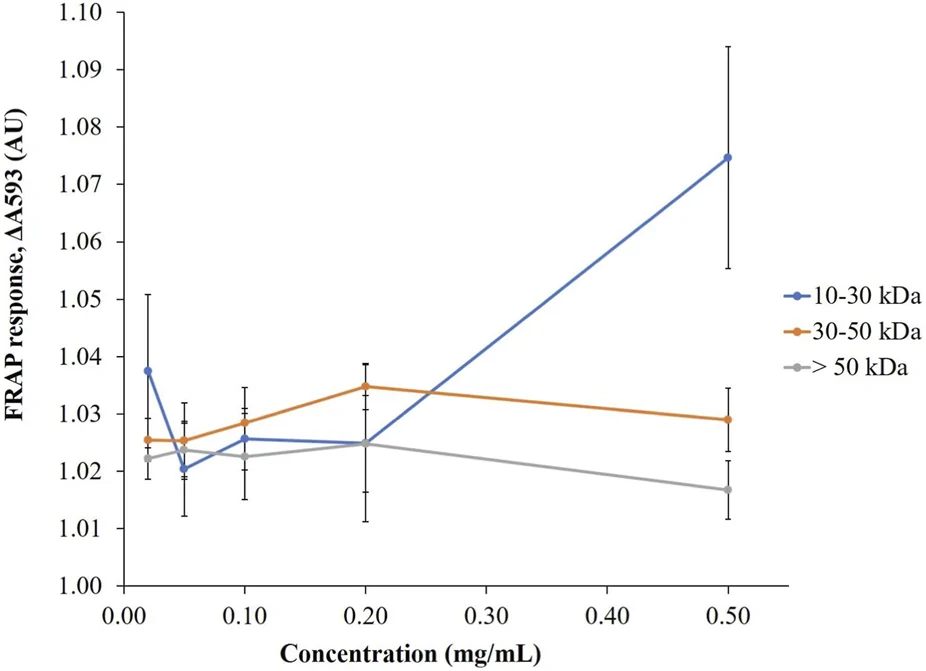
Ferric-reducing activity of the peptide fractions determined by the FRAP assay. The 10–30, 30–50, and >50 kDa peptide fractions were evaluated at concentrations of 0.02, 0.50, 0.10, 0.20, and 0.50 mg/mL. FRAP responses are expressed as blank-corrected absorbance at 593 nm (ΔA593, AU). Values are presented as mean ± SD (n = 4). Two-way ANOVA showed no significant effects of molecular-weight fraction, concentration, or their interaction (*p* > 0.05).

### 3.5 MTT assay

The peptide fractions were examined for their effects on HT-29 and HepG2 cell viability. Cells were treated with the three molecular-weight fractions (10–30, 30–50, and >50 kDa) at final concentrations of 0.1–0.8 mg/mL for 24 h. In HT-29 cells, the 10–30, 30–50, and >50 kDa fractions showed IC_50_ values of 0.634, 0.660, and 0.705 mg/mL, respectively (Figure 4A). The corresponding IC_50_ values in HepG2 cells were 0.761, 0.756, and 0.843 mg/mL, respectively (Figure 4B). The 10–30 kDa peptide fraction showed the lowest IC_50_ value in HT-29 cells and an IC_50_ value comparable to that of the 30–50 kDa fraction in HepG2 cells. Therefore, the 10–30 kDa peptide fraction was selected for further experiments.

**FIGURE 4:**
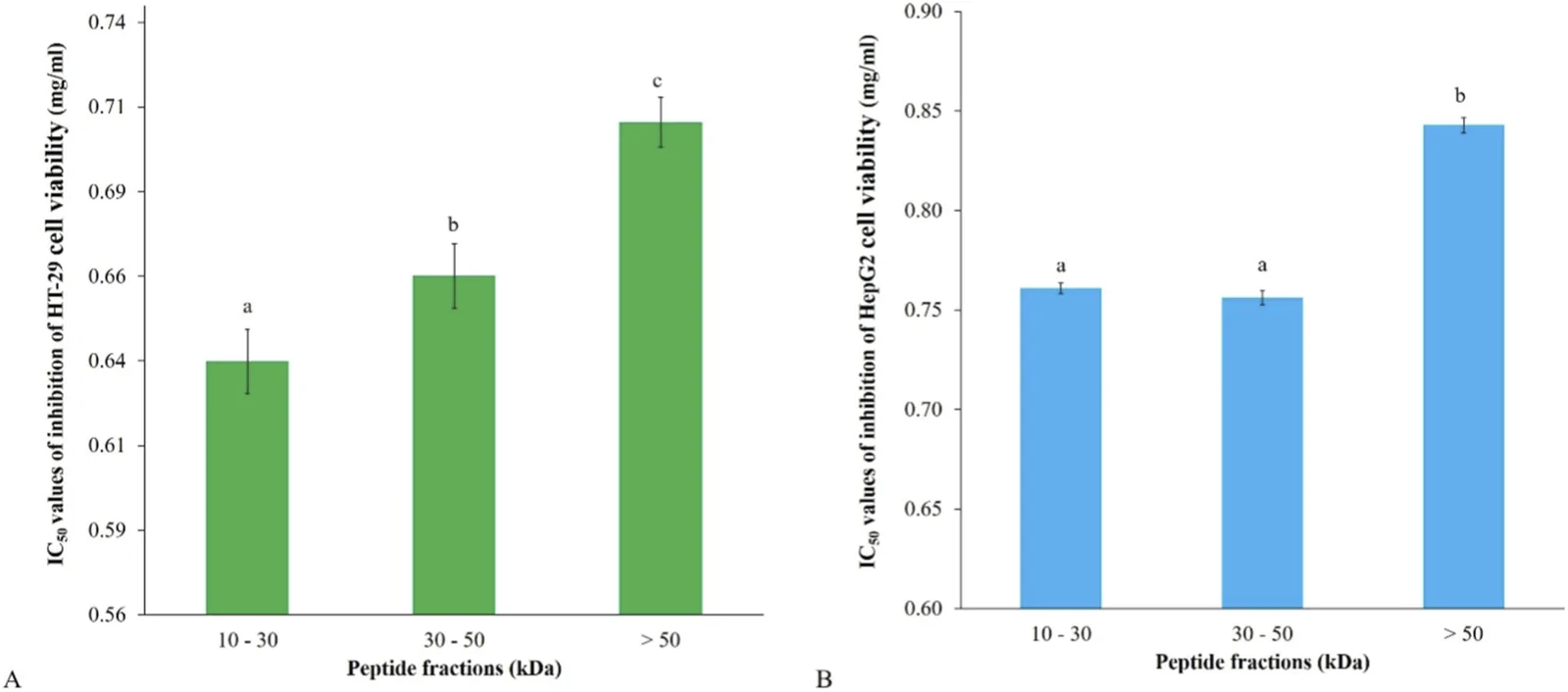
Effects of the peptide fractions on HT-29 and HepG2 cell viability. **(A)** Human colorectal adenocarcinoma (HT-29) cells and **(B)** human hepatocellular carcinoma (HepG2) cells were treated with the 10–30, 30–50, and >50 kDa peptide fractions for 24 h. Values are presented as mean ± SD from three independent experiments, each performed in triplicate. Different lowercase letters (a–c) indicate significant differences among groups (*p* < 0.05).

### 3.6 Cytotoxicity in non-cancerous cells

The cytotoxicity of the 10–30 kDa peptide fraction toward non-cancerous cells was evaluated using L929 fibroblast cells at concentrations corresponding to the IC_50_ values determined for HT-29 (0.634 mg/mL) and HepG2 (0.761 mg/mL) cells. L929 cell viability was 100.00% in the untreated control and 109.15% in the ascorbic acid-treated group. Treatment with the 10–30 kDa peptide fraction at 0.634 and 0.761 mg/mL resulted in cell viabilities of 109.85% and 96.40%, respectively (Figure 5). One-way ANOVA showed a significant difference among the treatment groups (*p* < 0.05). The 0.634 mg/mL treatment did not differ significantly from the ascorbic acid group (*p* > 0.05), whereas the 0.761 mg/mL treatment did not differ significantly from the untreated control (*p* > 0.05). The 0.634 mg/mL treatment showed significantly higher cell viability than the untreated control and the 0.761 mg/mL treatment (*p* < 0.05). Overall, L929 cells maintained high viability following exposure to the 10–30 kDa peptide fraction at both tested concentrations.

**FIGURE 5:**
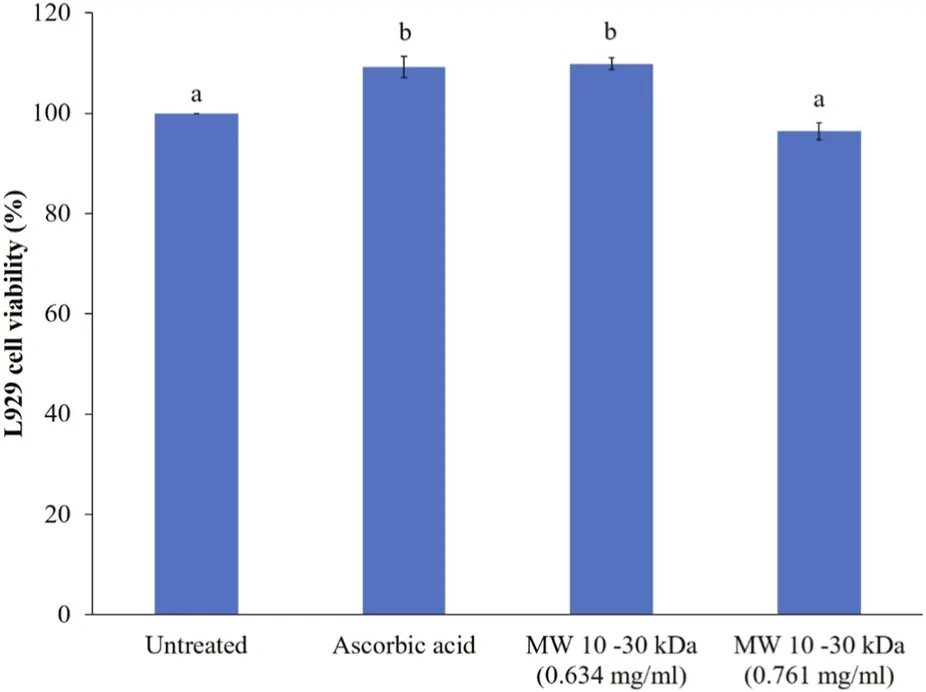
Viability of L929 fibroblast cells following treatment with the 10–30 kDa peptide fraction at concentrations corresponding to the IC_50_ values of HT-29 and HepG2 cells. Values are presented as mean ± SD. Different lowercase letters indicate significant differences among treatment groups (*p* < 0.05).

### 3.7 Determination of morphological features under an inverted light microscope

HT-29 and HepG2 cells were treated with the 10–30 kDa peptide fraction for 24 h, as presented in Figure 6. Untreated HT-29 and HepG2 cells retained their original morphology, with intact nuclei, relatively uniform cell size, and smooth cell membranes (Figures 6A,B). HT-29 and HepG2 cells treated with 1 nM paclitaxel are shown in Figures 6C,D. HT-29 cells were treated with the 10–30 kDa peptide fraction at 0.634 mg/mL (Figure 6E), whereas HepG2 cells were treated at 0.761 mg/mL (Figure 6F). Treatment resulted in morphological alterations, including cell shrinkage, rounding, membrane blebbing, and reduced cell-to-cell interactions. These morphological changes are consistent with apoptosis-associated cellular alterations.

**FIGURE 6:**
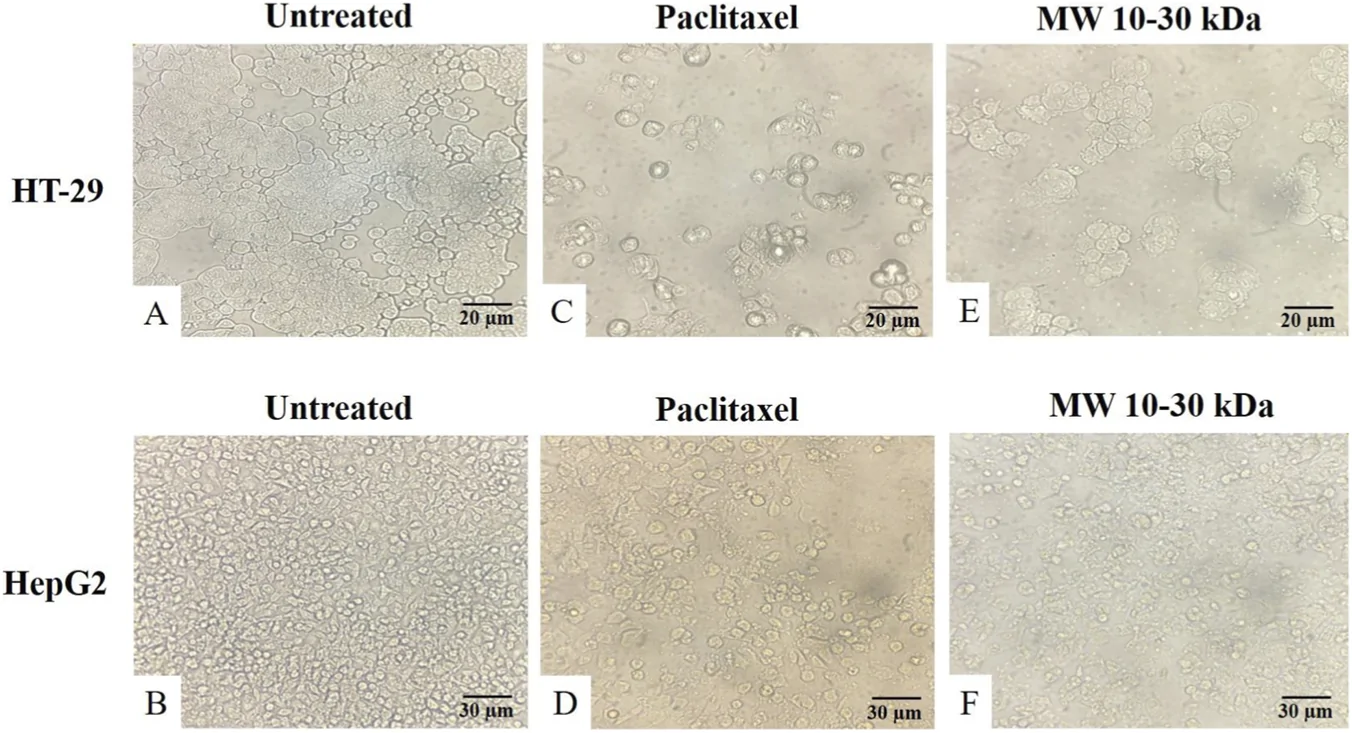
Morphological features of HT-29 and HepG2 cells observed under an inverted light microscope after 24 h of treatment. **(A)** Untreated HT-29 cells; **(B)** untreated HepG2 cells; **(C)** HT-29 cells treated with 1 nM paclitaxel; **(D)** HepG2 cells treated with 1 nM paclitaxel; **(E)** HT-29 cells treated with the 10–30 kDa peptide fraction at 0.634 mg/mL; and **(F)** HepG2 cells treated with the 10–30 kDa peptide fraction at 0.761 mg/mL. Images were acquired at ×200 magnification.

### 3.8 Assessment of apoptosis-associated morphological features using AO/PI staining

Untreated HT-29 and HepG2 cells appeared healthy and viable, with bright green fluorescence and intact nuclear structures (Figures 7A,B). HT-29 and HepG2 cells treated with paclitaxel are shown in Figures 7C,D. The 10–30 kDa peptide fraction at 0.634 mg/mL was tested on HT-29 cells (Figure 7E), whereas the fraction at 0.761 mg/mL was tested on HepG2 cells (Figure 7F). AO/PI staining revealed changes in fluorescence patterns and cellular morphology consistent with apoptosis-associated changes. Greenish-orange cells with membrane blebbing and orange-to red-stained cells were observed following treatment. An increased proportion of orange-to red-stained cells was visually observed in cells treated with the 10–30 kDa peptide fraction compared with untreated cells. These observations provide morphological evidence consistent with apoptosis.

**FIGURE 7:**
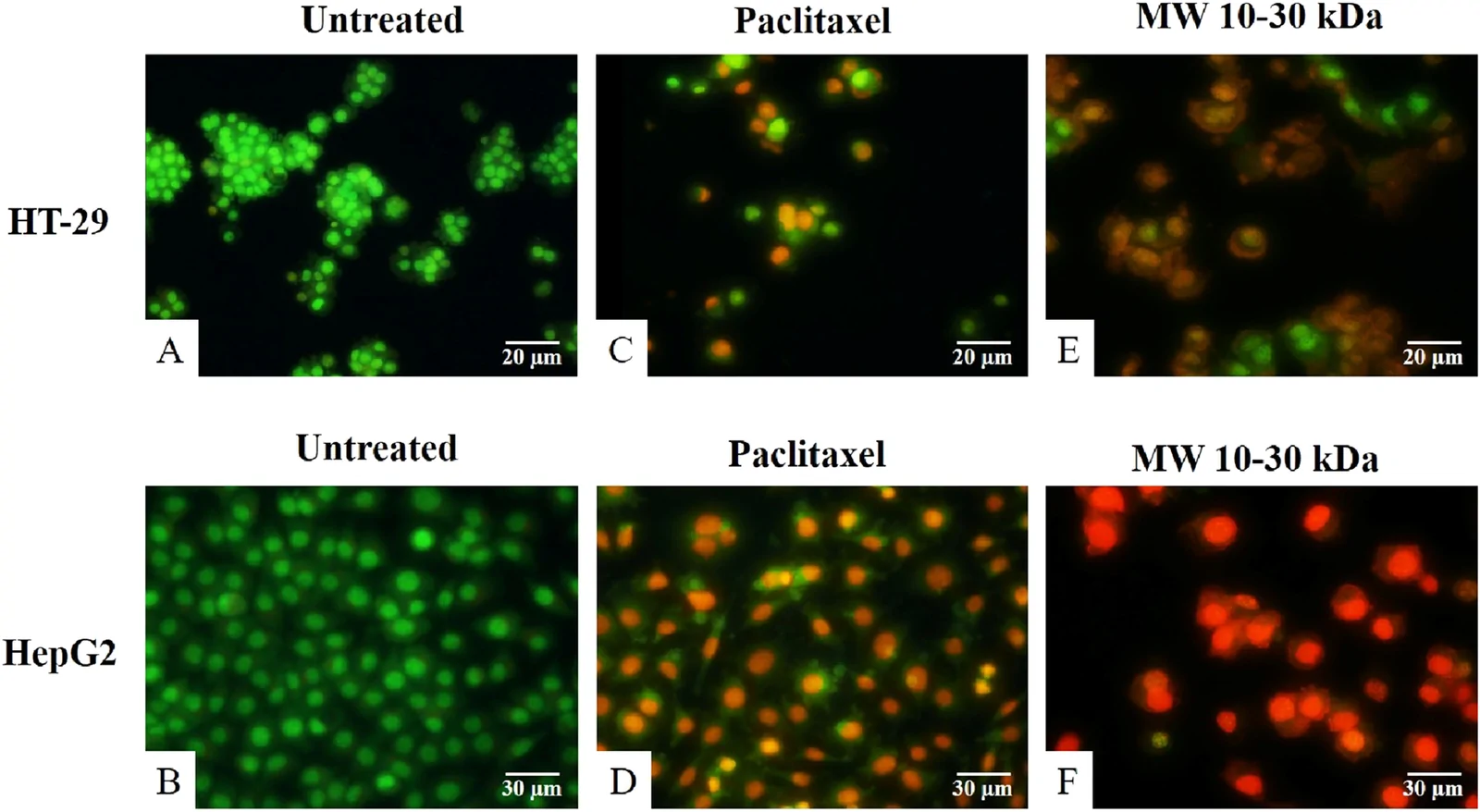
Apoptosis-associated morphological features of HT-29 and HepG2 cells evaluated using AO/PI double staining after 24 h of treatment. **(A)** Untreated HT-29 cells; **(B)** untreated HepG2 cells; **(C)** HT-29 cells treated with paclitaxel; **(D)** HepG2 cells treated with paclitaxel; **(E)** HT-29 cells treated with the 10–30 kDa peptide fraction at 0.634 mg/mL; and **(F)** HepG2 cells treated with the 10–30 kDa peptide fraction at 0.761 mg/mL. Images were acquired at ×400 magnification.

### 3.9 Assessment of nuclear chromatin condensation using DAPI staining

HT-29 and HepG2 cells were treated with the 10–30 kDa peptide fraction for 24 h and examined using DAPI staining. Untreated HT-29 and HepG2 cells remained intact and showed relatively uniform nuclear morphology (Figures 8A,B). HT-29 and HepG2 cells treated with paclitaxel are shown in Figures 8C,D. The 10–30 kDa peptide fraction was applied to HT-29 and HepG2 cells at concentrations of 0.634 and 0.761 mg/mL, respectively (Figures 8E,F). DAPI staining revealed nuclear condensation and fragmentation in treated cells compared with untreated cells. These nuclear alterations are consistent with apoptosis-associated morphological changes.

**FIGURE 8:**
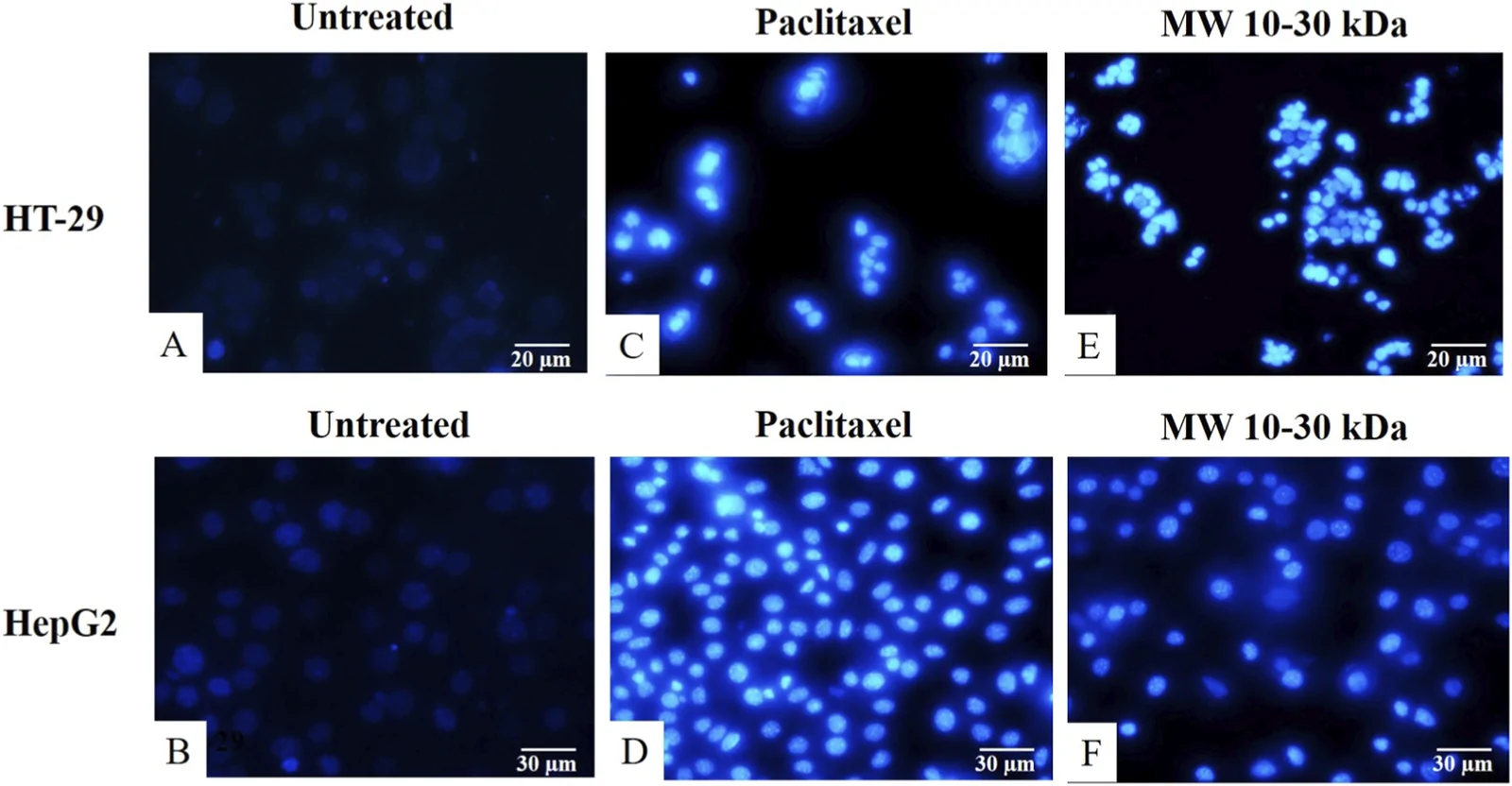
Nuclear morphological changes in HT-29 and HepG2 cells evaluated by DAPI staining after 24 h of treatment. **(A)** Untreated HT-29 cells; **(B)** untreated HepG2 cells; **(C)** HT-29 cells treated with paclitaxel; **(D)** HepG2 cells treated with paclitaxel; **(E)** HT-29 cells treated with the 10–30 kDa peptide fraction at 0.634 mg/mL; and **(F)** HepG2 cells treated with the 10–30 kDa peptide fraction at 0.761 mg/mL. Images were acquired at ×400 magnification.

### 3.10 Assessment of DNA fragmentation by agarose gel electrophoresis

DNA fragmentation patterns were examined in HT-29 and HepG2 cells following treatment with the 10–30 kDa peptide fraction. Untreated HT-29 cells showed non-fragmented DNA (Figure 9A, Lane 1), whereas paclitaxel-treated HT-29 cells displayed fragmented DNA (Figure 9A, Lane 2). HT-29 cells treated with the 10–30 kDa peptide fraction at 0.634 mg/mL for 24 h showed DNA fragmentation and smearing (Figure 9A, Lane 3).

**FIGURE 9:**
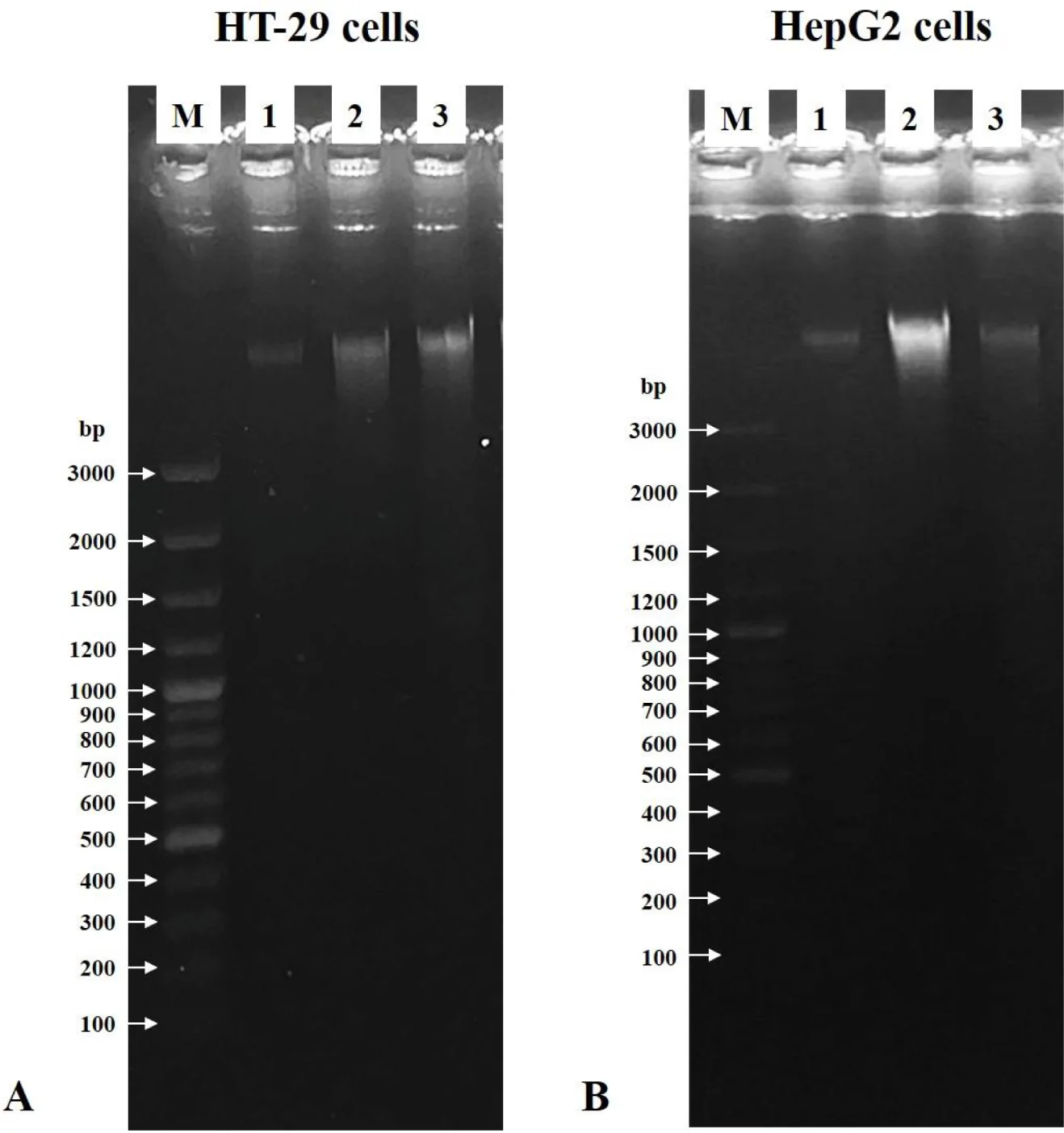
DNA fragmentation patterns of HT-29 and HepG2 cells following 24 h of treatment. **(A)** HT-29 cells: Lane M, GeneRuler 100 bp Plus DNA Ladder; Lane 1, untreated cells; Lane 2, cells treated with 1 nM paclitaxel; and Lane 3, cells treated with the 10–30 kDa peptide fraction at 0.634 mg/mL. **(B)** HepG2 cells: Lane M, GeneRuler 100 bp Plus DNA Ladder; Lane 1, untreated cells; Lane 2, cells treated with 1 nM paclitaxel; and Lane 3, cells treated with the 10–30 kDa peptide fraction at 0.761 mg/mL.

Similarly, untreated HepG2 cells showed non-fragmented DNA (Figure 9B, Lane 1), whereas paclitaxel-treated HepG2 cells showed fragmented DNA (Figure 9B, Lane 2). HepG2 cells treated with the 10–30 kDa peptide fraction at 0.761 mg/mL for 24 h showed DNA fragmentation and smearing (Figure 9B, Lane 3). The observed DNA fragmentation and smearing provide additional evidence of treatment-associated DNA degradation.

### 3.11 Scanning electron microscope

The surface morphological features of HT-29 and HepG2 cells were examined using high-vacuum scanning electron microscopy (SEM). Untreated HT-29 and HepG2 cells (Figures 10A,B) generally showed intact cell membranes, cell-to-cell interactions, and microvilli-like structures. HT-29 and HepG2 cells treated with paclitaxel are shown in Figures 10C,D. HT-29 cells treated with the 10–30 kDa peptide fraction at 0.634 mg/mL (Figure 10E) and HepG2 cells treated at 0.761 mg/mL (Figure 10F) showed reduced cell-to-cell interactions and alterations in microvilli-like structures. These surface morphological alterations were consistent with cellular damage and apoptosis-associated morphological changes observed in the other morphological and staining assays.

**FIGURE 10:**
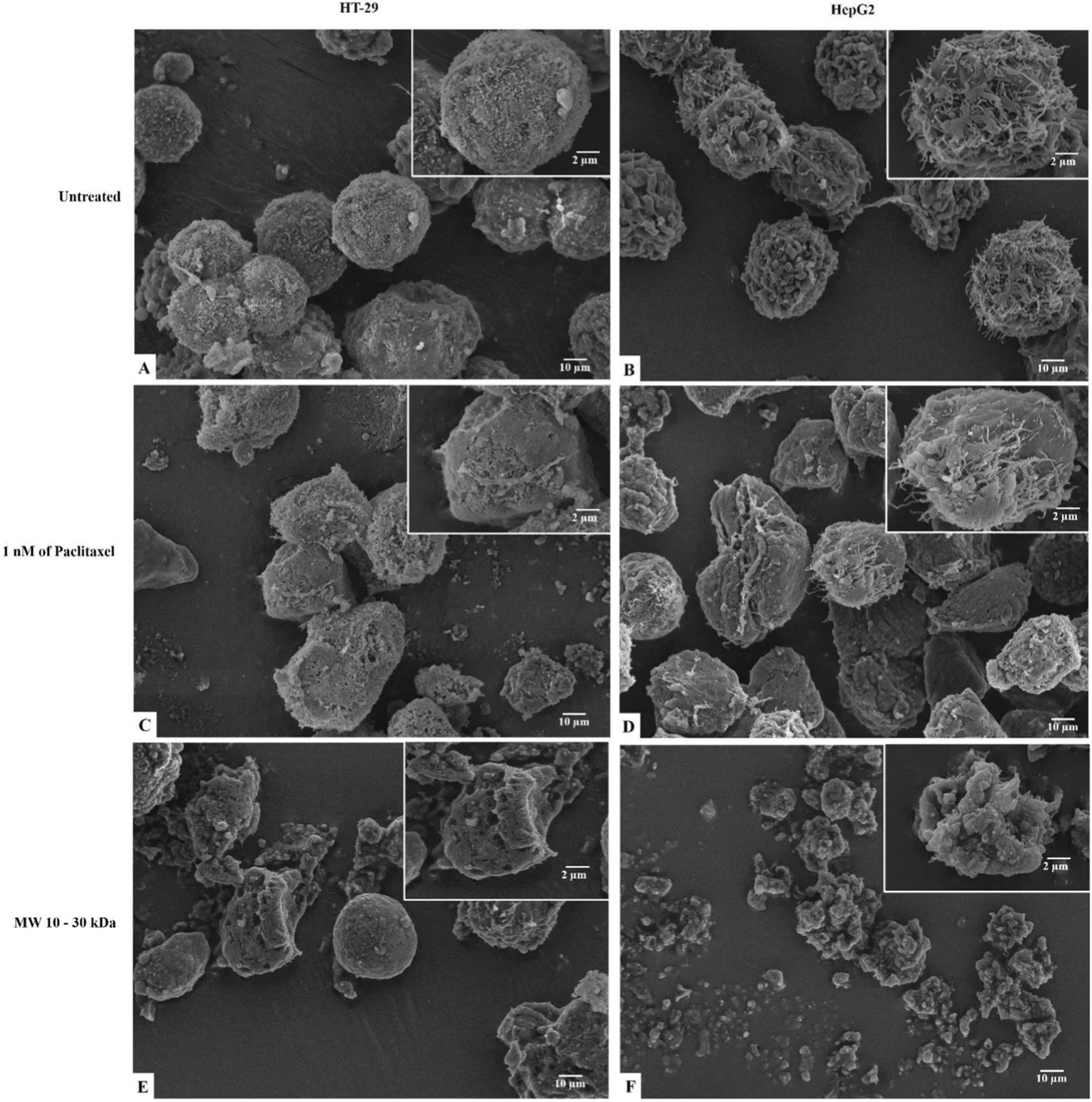
Surface morphological features of HT-29 and HepG2 cells examined by scanning electron microscopy (SEM). **(A)** Untreated HT-29 cells; **(B)** untreated HepG2 cells; **(C)** HT-29 cells treated with 1 nM paclitaxel; **(D)** HepG2 cells treated with 1 nM paclitaxel; **(E)** HT-29 cells treated with the 10–30 kDa peptide fraction at 0.634 mg/mL; and **(F)** HepG2 cells treated with the 10–30 kDa peptide fraction at 0.761 mg/mL. SEM images were acquired at ×5,000 and ×20,000 magnifications with an accelerating voltage of 7 kV.

### 3.12 Scratch assay for cell migration

Untreated HT-29 and HepG2 cells exhibited cell migration values of 47.73% ± 10.13% and 35.08% ± 3.01%, respectively, after 24 h. In comparison, paclitaxel-treated HT-29 and HepG2 cells showed cell migration values of 35.71 ± 4.08% and 30.51%± 0.40%, respectively, whereas cells treated with the 10–30 kDa peptide fraction showed values of 32.14% ± 6.07% and 24.66% ± 7.72%, respectively. Overall, the treated groups showed numerically lower cell migration values than the corresponding untreated controls (Figure 11A).

**FIGURE 11:**
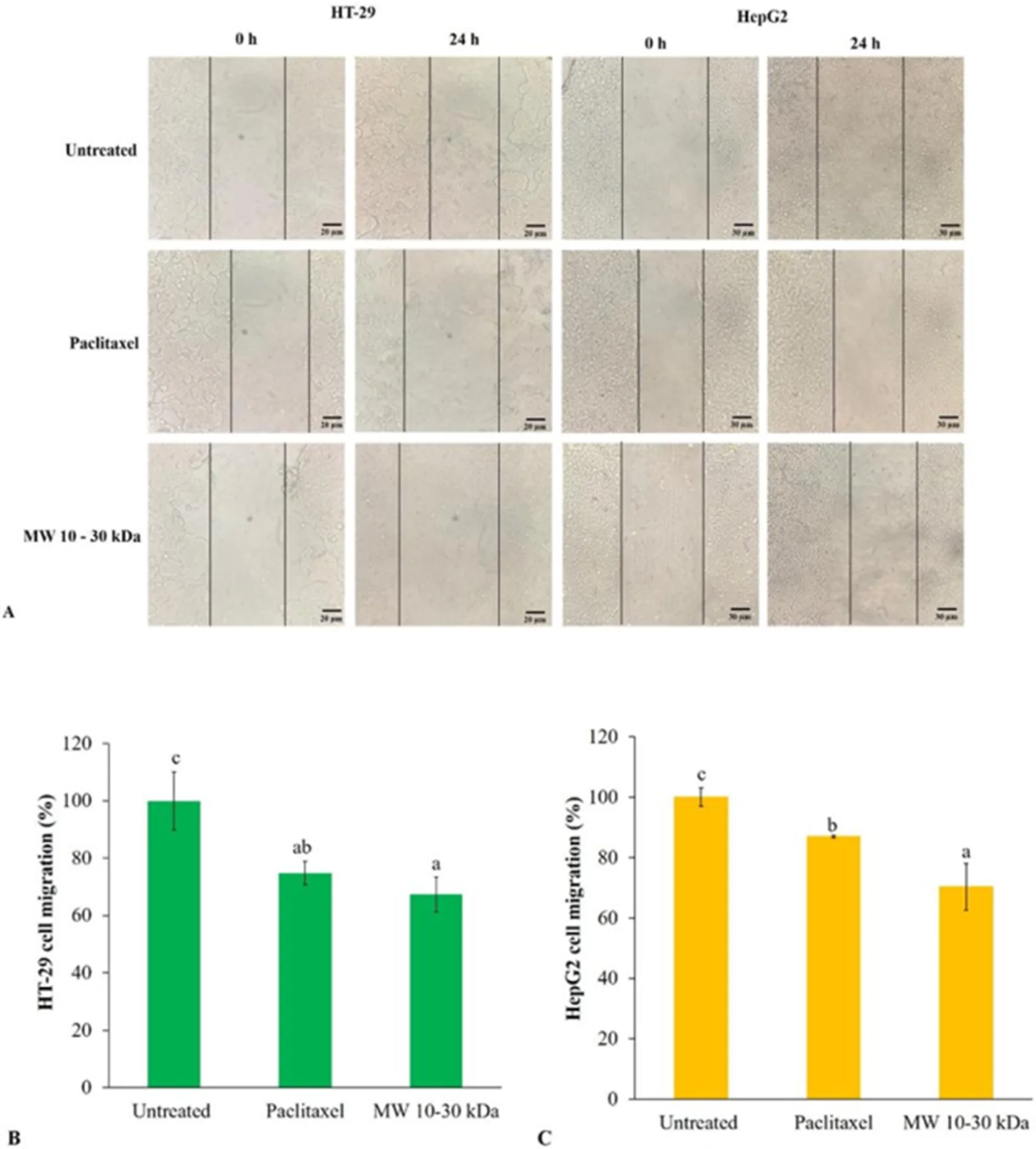
**(A)** Apparent cell migration of HT-29 and HepG2 cells evaluated using the scratch assay after 24 h of treatment. **(B)** HT-29 cells treated with the 10–30 kDa peptide fraction at 0.634 mg/mL and **(C)** HepG2 cells treated with the 10–30 kDa peptide fraction at 0.761 mg/mL, corresponding to their respective IC_50_ values determined by the MTT assay. Paclitaxel served as the positive control. Cell migration was evaluated based on changes in the scratched area. Values are presented as mean ± SD. Different lowercase letters **(A–C)** indicate significant differences among groups (*p* < 0.05). Images were acquired at ×200 magnification.

For relative comparison, the cell migration values of the untreated controls were normalized to 100%. The corresponding relative cell migration values for paclitaxel and the 10–30 kDa peptide fraction were 74.81% and 67.34%, respectively, in HT-29 cells (Figure 11B), and 86.97% and 70.29%, respectively, in HepG2 cells (Figure 11C). However, because the 10–30 kDa peptide fraction was tested at its IC_50_ concentration, the observed reduction in cell migration may reflect combined effects on cell proliferation and cell movement. Therefore, these findings indicate reduced apparent cell migration under the tested conditions rather than a specific antimigratory effect.

## 4 Discussions

*Amynthas arenulus* earthworm proteins were hydrolyzed with Alcalase® under conditions optimized using RSM. Alcalase is a protease capable of hydrolyzing protein molecules into smaller peptide components (García et al., 2022). Alcalase has been reported to cleave peptide bonds involving Glu, Met, Leu, Tyr, Lys, and Gln residues on the carboxyl side (Adamson and Reynolds, 1996). In this manner, Alcalase can be used to produce protein hydrolysates containing smaller peptides (Tacias-Pascacio et al., 2020). Compared with several other proteases, Alcalase has been reported to provide effective protein hydrolysis and relatively high degrees of hydrolysis (Forghani et al., 2012). In the present study, hydrolysis of *A. arenulus* earthworm proteins was optimal at an E/S ratio of 1.5% (v/v), a hydrolysis time of 2 h, pH 8.0, and 60 °C. Under these conditions, the highest TCA-soluble peptide index (DH-TCA) was 61.25%. Similarly, *Eisenia andrei* earthworm protein was reported to show a DH of 92% under optimized conditions of an E/S ratio of 1.77%, pH 9.5, 25 °C, 200 rpm, and a hydrolysis time of 2.25 h (Rodrigues et al., 2017), whereas eel (*Monopterus* sp.) protein showed a DH of 15.01% at pH 9.0, 55.76 °C, and an enzyme concentration of 1.80% (Jamil et al., 2016). Differences in reported hydrolysis values may be related to the protein source, enzyme concentration, hydrolysis conditions, and analytical method used. Because the present study used the TCA-soluble peptide method, the DH-TCA value represents the proportion of TCA-soluble peptide material and serves as an indirect operational index of proteolysis rather than a direct measurement of peptide-bond cleavage.

The protein hydrolysates from *A. arenulus* earthworms showed recovery of peptide material following simulated gastrointestinal digestion. Pepsin and pancreatin were used to simulate gastric and intestinal digestion, respectively. Similarly, peptide materials from mealworm (*Tenebrio molitor*) larvae (Mancini et al., 2021) and spotted babylon snails (*Babylonia areolata*) (Petsantad et al., 2020) have been investigated under simulated gastrointestinal conditions. Pepsin, which is involved in gastric protein digestion, is an endopeptidase capable of cleaving peptide bonds involving several amino acids, particularly hydrophobic and aromatic residues (Kageyama, 2002; Petsantad et al., 2020) including Phe, Trp, and Tyr (Fruton, 1970).

Pancreatin contains several digestive enzymes, including proteases capable of cleaving peptide bonds associated with basic amino acid residues such as Lys and Arg (Petsantad et al., 2020). Peptide structure and terminal amino acid residues can influence stability during gastrointestinal digestion. Previous studies have suggested that some bioactive peptides may exhibit resistance to gastrointestinal proteolysis (He et al., 2018), while gastrointestinal digestion can also increase the amounts of free amino acids and low-molecular-weight peptides (Abrahamse et al., 2022). However, gastrointestinal stability does not necessarily indicate intestinal absorption or bioavailability, and these properties require further evaluation using appropriate models. In the present study, peptide material showed a gastric recovery of 56.83% and an intestinal recovery of 90.10%. Following simulated gastrointestinal digestion, the recovered material was further separated into apparent molecular-weight fractions, including the 10–30 kDa fraction. These recovery values represent the gravimetric recovery of soluble peptide material after each digestion phase rather than the proportion of intact peptides resistant to gastrointestinal proteolysis.

The antioxidant properties of the peptide fractions were evaluated using DPPH^•^, ABTS^•+^, and FRAP assays. The DPPH^•^ assay assesses the ability of antioxidants to scavenge DPPH radicals, which are reduced upon reaction with hydrogen- or electron-donating compounds (Brand-Williams et al., 1995). The ABTS^•+^ assay evaluates the reduction of the ABTS radical cation in the presence of hydrogen-donating antioxidants (Re et al., 1999). Meanwhile, the FRAP assay evaluates ferric-reducing activity through the reduction of the Fe^3+^-TPTZ complex to its ferrous form, Fe^2+^-TPTZ (Benzie and Strain, 1996). Natural antioxidant compounds and peptides have been investigated for their ability to reduce oxidative stress and scavenge reactive species (Chi et al., 2015; Liu et al., 2020). In the present study, the 10–30 kDa peptide fraction showed estimated IC_50_ values of 3.18 mg/mL for DPPH and 0.79 mg/mL for ABTS radical-scavenging activity. For the FRAP assay, ferric-reducing activity was expressed as blank-corrected absorbance at 593 nm (ΔA593) rather than as an IC_50_ value. The 10–30 kDa fraction showed ΔA593 values ranging from approximately 1.020–1.046 AU across the tested concentrations. Previous studies have reported that molecular size can influence the antioxidant activity of protein hydrolysates and peptides (Yuan et al., 2017). Similarly, Tra catfish peptides with molecular weights of 10–30 kDa demonstrated DPPH radical-scavenging activity, with a reported IC_50_ value of 8.01 mg/mL (Vo et al., 2017), while PAAE from *A. arenulus* have previously shown DPPH and ABTS radical-scavenging activities (Wasunan et al., 2022).

Furthermore, the 10–30 kDa peptide fraction reduced HT-29 and HepG2 cell proliferation *in vitro*. The fraction was also evaluated in non-cancerous L929 fibroblast cells, which maintained relatively high viability at the tested concentrations, suggesting lower cytotoxicity toward L929 cells under these conditions. However, because a complete concentration-response analysis was not performed in L929 cells, these findings provide only preliminary evidence of differential cytotoxicity rather than definitive cancer-cell selectivity. Similarly, cytotoxic or antiproliferative activities have been reported for earthworm-derived proteins. Purified proteins from *Aporrectodea longa* earthworm (MW 42 kDa) inhibited the proliferation of HT-29, HepG2, and MCF-7 cells, with reported IC_50_ values of 0.06, 0.08, and 0.09 mg/mL, respectively (Alotaibi, 2022). Mucus proteins from *Eudrilus eugeniae* earthworm (MW 8–60 kDa) and *Perionyx excavatus* earthworm (MW 12–100 kDa) were also reported to inhibit HepG2 cell proliferation, with IC_50_ values of 0.14 and 0.17 mg/mL, respectively (Prem-U-Domkit et al., 2017). PAAEs from *A. arenulus* earthworm were also reported to inhibit HepG2 cell proliferation, with an IC_50_ value of 0.54 mg/mL (Wasunan et al., 2022).

In this study, apoptosis-associated morphological changes were observed and described. Treated cells were stained with AO/PI and DAPI fluorescence dyes and were also examined under SEM to assess apoptosis-associated cellular changes. Apoptosis is a process characterized by morphological changes such as microvilli loss, chromatin condensation, membrane blebbing, and the formation of apoptotic bodies (Cheng et al., 2016). Furthermore, DNA fragmentation was examined using agarose gel electrophoresis (Suganya et al., 2019). In this study, HT-29 and HepG2 cells exposed to the 10–30 kDa peptide fraction showed reduced cell viability/proliferation together with apoptosis-associated cellular changes. However, the assays used in the present study provide supportive morphological and DNA-fragmentation evidence rather than direct confirmation of a specific apoptotic pathway, because mechanistic analyses such as Annexin V/PI flow cytometry, caspase activation, and apoptosis-related molecular markers were not performed.

One possible explanation reported in the literature is related to differences in membrane characteristics between normal and cancer cells. The outer leaflet of normal cell membranes generally has a more neutral net charge, whereas cancer cell membranes may exhibit a relatively negative surface charge that can facilitate interactions with cationic peptides (Hwang et al., 2022). However, the charge characteristics and membrane interactions of the peptide fraction used in the present study were not determined; therefore, this mechanism cannot be directly inferred from the present findings (Lou et al., 2015). The surface morphological features of HT-29 and HepG2 cells were investigated using SEM. SEM revealed cell-to-cell interactions, matrix-like materials, and microvilli-like structures. Microvilli-like structures have previously been reported on the surfaces of both HT-29 and HepG2 cells (Solmi et al., 2008; Lou et al., 2015). Longer and denser microvilli have been associated with increased invasive, metastatic, and proliferative capacity (Han et al., 2021), while microvilli are also involved in the transport of metabolites and fluids across cell surfaces (Lange, 2011; Han et al., 2021).

Previous studies have proposed that some anticancer peptides may affect cancer-cell membranes through mechanisms such as pore formation, membrane lysis, membrane thinning, membrane dissolution, or lipid–peptide domain formation (Wang et al., 2017; Chiangjong et al., 2020). However, the molecular components and mechanisms responsible for the effects observed in the present study were not directly investigated; therefore, these mechanisms can only be considered possible explanations based on previous reports. Cellular adhesion between adjacent cells and the presence of microvilli have also been used as morphological characteristics for evaluating cellular responses to treatment (Solmi et al., 2008). Treatment may also affect the plasma membrane and cytoskeletal organization of cells (Solmi et al., 2008). Overall, the morphological, fluorescence-staining, and DNA-fragmentation observations in the present study were consistent with apoptosis-associated cellular changes. Nevertheless, additional mechanistic analyses are required to confirm the pathways responsible for these effects.

Cell migration involves both single-cell and collective-cell migration (Grada et al., 2017). Collective cell migration is influenced by cell polarization, coordination of cytoskeletal activity, cell–cell adhesion, and chemical and mechanical signals (Friedl and Gilmour, 2009). The scratch assay is an *in vitro* method commonly used to investigate cell migration and cell-movement-related processes (Bobadilla et al., 2019). When an artificial gap or “scratch” is created in a confluent cell monolayer, cells at the edge of the gap move and proliferate toward the open area until cell–cell contacts are re-established (Liang et al., 2007). In this study, lower apparent cell-migration values were observed in HT-29 and HepG2 cells treated with the 10–30 kDa peptide fraction compared with untreated cells. However, because the fraction was evaluated at its IC_50_ concentration, the observed reduction may reflect combined effects on cell proliferation and cell movement. Similar reductions in cell migration have been reported for peptides derived from unicorn leatherjacket fish (*Aluterus monoceros*) in COLO 320 cells (Kumar et al., 2019). Cellular migration is an important component of cancer metastatic progression (Bravo-Cordero et al., 2012; Bobadilla et al., 2019).

The present findings demonstrate *in vitro* antiproliferative activity and apoptosis-associated cellular changes in HT-29 and HepG2 cells following treatment with the 10–30 kDa peptide fraction. Nevertheless, further studies involving molecular characterization of the active components, mechanistic analyses, appropriate non-cancerous human cell models, and *in vivo* efficacy and safety evaluations are required before its potential therapeutic relevance can be established.

## 5 Conclusion

In this study, the optimal hydrolysis conditions for *A. arenulus* earthworm proteins were determined using RSM. The resulting protein hydrolysates showed recovery of peptide material following simulated gastrointestinal digestion. The peptide fractions exhibited antioxidant activity, including radical-scavenging and ferric-reducing activities. Among the fractions examined, the 10–30 kDa peptide fraction showed notable biological activity and was selected for further cellular assays. In HT-29 and HepG2 cells, treatment with the 10–30 kDa peptide fraction was associated with reduced cell viability and apoptosis-associated morphological changes, as observed using inverted light microscopy, AO/PI and DAPI staining, and SEM, together with DNA fragmentation. The fraction was also associated with reduced wound closure under the tested conditions. However, because the scratch-wound assay was performed at IC_50_ concentrations, the reduced wound closure may reflect combined effects on cell proliferation and cell movement. Overall, the present findings indicate *in vitro* antioxidant and antiproliferative activities, together with apoptosis-associated cellular changes, of the *A. arenulus* peptide fractions. These findings provide preliminary evidence of their biological activities. Further *in vitro* studies, including mechanistic investigations and evaluation in appropriate non-cancerous human cell models, as well as *in vivo* efficacy and safety evaluations, toxicity assessment, bioavailability studies, and molecular characterization, are warranted before potential medical or functional food applications can be considered.

## Data Availability

The original contributions presented in the study are included in the article/supplementary material, further inquiries can be directed to the corresponding author.
